# Timing matters: the impact of response measures on COVID-19-related hospitalization and death rates in Germany and Switzerland

**DOI:** 10.1186/s41937-020-00054-w

**Published:** 2020-08-26

**Authors:** Martin Huber, Henrika Langen

**Affiliations:** University of Fribourg, Bd. de Pérolles 90, Fribourg, 1700 Switzerland

**Keywords:** COVID-19, Pandemic, Social distancing, Lockdown, Treatment effect, Synthetic control, I18, I12, H12

## Abstract

We assess the impact of the timing of lockdown measures implemented in Germany and Switzerland on cumulative COVID-19-related hospitalization and death rates. Our analysis exploits the fact that the epidemic was more advanced in some regions than in others when certain lockdown measures came into force, based on measuring health outcomes relative to the region-specific start of the epidemic and comparing outcomes across regions with earlier and later start dates. When estimating the effect of the relative timing of measures, we control for regional characteristics and initial epidemic trends by linear regression (Germany and Switzerland), doubly robust estimation (Germany), or synthetic controls (Switzerland). We find for both countries that a relatively later exposure to the measures entails higher cumulative hospitalization and death rates on region-specific days after the outbreak of the epidemic, suggesting that an earlier imposition of measures is more effective than a later one. For Germany, we further evaluate curfews (as introduced in a subset of states) based on cross-regional variation. We do not find any effects of curfews on top of the federally imposed contact restriction that banned groups of more than 2 individuals.

## Introduction

This paper assesses how the timing of the lockdown measures implemented in Switzerland and Germany affects the development of cumulative COVID-19-related hospitalization and death rates. In both countries, the federal governments implemented extensive lockdown measures, including the closure of non-essential shops, schools, childcare centers, cafes, bars, and restaurants. In Germany, these measures were further enhanced with a ban on gatherings with more than two people decided at federal level and curfews implemented in several states. With the measures in place for some weeks, both countries report a flattening of the COVID-19 epidemic curve. This alone, however, does not necessarily exclusively reflect the impact of the measures, but likely also general time trends in the spread of the virus. For this reason, this study aims to provide evidence about the causal effects of the German and Swiss measures by exploiting variation (i) in their relative timing due the fact that the epidemic was more advanced in some regions than in others when certain measures came into force and (ii) across regions due to the fact that some measures were only introduced in a subset of regions.

A range of studies on the impact of COVID-19 response measures focus on predicting the development of the pandemic in terms of infections, hospitalizations, or death rates based on simulating the spread of the virus and calibrating the model as a function of the measures. For instance, Koo et al. (2020) provide a simulation study on the COVID-19 outbreak in Singapore and model the development of COVID-19 infections under four potential intervention scenarios. Likewise, Bicher et al. (2020) developed an agent-based simulation model to predict the development of infections under different scenarios of lockdown timing and exit strategies out of the lockdown in Austria, finding that delaying the lockdown by 1 week would have translated into an increase of infections by 4 times. Donsimoni et al. (2020) simulate the effect of lockdown timing and duration on the rate of COVID-19 infections and the expected end date of the epidemic in Germany. The study suggests that a complete lift of measures on April 20 would have borne the risk of increasing infection rates. The authors further advise to adopt exit strategies and policies that differ across regions in order to learn about which measures are most effective for containing the epidemic while reducing social and economic costs.

In contrast to such simulations, in which empirical data serve for calibrating parameters in prediction models, a growing literature applies policy evaluation methods as outlined in Imbens and Wooldridge (2009) to assess the effectiveness of lockdown measures based on variation across regions and over time. Qiu et al. (2020) for instance investigate the influence of socioeconomic factors and COVID-19 response measures on transmission dynamics in China, finding that measures at a local level have a larger impact on the epidemic curve than restricting population flows between cities. Juranek and Zoutman (2020) use an event study approach to assess the effect of the lockdown measures of Denmark and Norway on hospitalizations based on a comparison with Sweden whose measures are comparably lenient. Results suggest that the peak number of hospitalizations would have more than doubled in Denmark and Norway had they followed Sweden’s strategy.

Dave et al. (2020) use a difference-in-differences approach to evaluate lockdown measures (namely shelter in place orders) in the USA by exploiting variation in responses across states and over time. As a consequence of the measures, they find an important increase (of 5–10%) in the rate at which state residents remained in their homes full-time as well as substantial reductions in cumulative COVID-19 cases (44% after 3 weeks),^1^ with early adopting states with a high population density benefiting most. See also Fowler et al. (2020) for a related difference-in-differences strategy for the USA that suggests reductions in infections, too, as well as in fatalities. Results in Friedson et al. (2020), who use a synthetic control approach to analyze the measures’ effectiveness in California, and Dave et al. (2020), who evaluate the impact of the measures implemented in Texas in an event study framework, point in the same direction. Weber (2020) exploits regional differences in the timing of measures in Germany finding that school closures, prohibition of mass events, and gathering bans and curfews played a major role in reducing the number of confirmed infections, while border closures and shutdowns of the service and retail sector did not show a significant effect. Studies on the impact of face mask requirements in public transport, retailers, and public businesses find evidence for a reduction in the spread of the virus through such requirements, see, for example, Mitze et al. (2020) for a synthetic control study on German data and Chernozhukov et al. (2020), who assess the impact of such requirements in the USA within a causal framework that allows for both direct effects of COVID-19 response measures and indirect effects through behavioral changes.

Askitas et al. (2020) apply an event study design to assess a range of different response measures across 135 countries and find that canceling public events and restricting gatherings reduce new infections more effectively than mobility restrictions like international travel controls. This is in line with Bonardi et al. (2020) who consider first difference and AR(1) models based on 184 countries and conclude that lockdown measures generally reduce confirmed infections and fatalities (and even more so if imposed rather earlier than later), while border closures do not show important effects. Findings in Banholzer et al. (2020), a study on 20 Western countries in a Bayesian framework, suggest that venue closures and gathering bans are most effective in reducing infections but also attest a significant effect of border closures.

Our paper contributes to this growing literature by analyzing COVID-19-related hospitalizations and death rates across administrative units over time, namely across counties in the case of Germany and across cantons in the case of Switzerland. We estimate the effect of the relative timing of lockdown measures based on measuring health outcomes relative to the region-specific start of the epidemic and comparing outcomes across regions with earlier and later start dates. The start date is defined as the day on which the confirmed regional infections per 10,000 inhabitants exceed 1 for the first time. In the analysis, we control for regional characteristics (population size and density, age structure, and GDP per capita), initial trends of the epidemic (median age of confirmed infections and initial growth rate of confirmed infections), and other policies selectively introduced prior to the major lockdowns (e.g., a ban on visits to hospitals and retirement homes in some regions).

Linear regression estimates suggest that for both Switzerland (which also includes the Principality of Liechtenstein as data point) and Germany, a relatively later exposure to the measures entails higher cumulative hospitalization and death rates on sufficiently advanced region-specific days after the outbreak of the epidemic. This suggests that an earlier imposition of measures is more effective than a later one w.r.t. our health outcomes, which is in line with findings in Amuedo-Dorantes et al. (2020) on the effect of lockdown timing on COVID-19-related deaths in Spain. For Germany with its substantially larger number of observations, we also estimate the effect of the relative timing based on doubly robust (DR) estimation, see Robins et al. (1994) and Robins and Rotnitzky (1995), which is a more flexible approach than exclusively relying on a linear outcome model. For Switzerland, we also consider the synthetic control method, see Abadie and Gardeazabal (2003) and Abadie et al. (2010), to assess for two selected cantons with a relatively late exposure what their counterfactual outcomes would have been under an earlier exposure. Both the DR and synthetic control methods corroborate the findings of the linear regression. For Germany only, we also evaluate the effect of curfews that were introduced by a subset of German states in addition to the federal lockdown measures and bans of gatherings with more than two individuals. Exploiting this cross-sectional variation while controlling for observed characteristics, neither linear regression nor DR estimation suggests that curfews further reduce hospitalizations and fatalities under the lockdown measures already in place, which is in line with the findings in Bonardi et al. (2020) and Banholzer et al. (2020). Apart from this assessment of the impact of curfews on COVID-19-related death rates, our analysis does not inform about the effectiveness of single social distancing measures implemented as part of the lockdown in Germany and Switzerland. Further, a cost-effectiveness assessment of COVID-19 response measures, which is certainly of great importance for policy makers, is beyond the scope of this paper, as the long-run social and economic consequences of the lockdown cannot be credibly assessed at the current stage.

The remainder of this paper is organized as follows. Section 2 provides an overview of the timeline of COVID-19 measures in Switzerland and Germany. Sections 3 and 4 describe the data and econometric methods used in the analyses. Section 5 presents and interprets the results. Section 6 concludes.

## Timeline of COVID-19 response measures

Both Germany and Switzerland are federal states with competencies in epidemic control partly belonging to the 26 cantons in Switzerland and the 16 federal states (Länder) in Germany. The German states themselves are comprised of all in all 401 counties (Kreise) which also have certain competencies in handling epidemic outbreaks. With competencies fragmented across the federal governments and subfederal authorities, not all measures were implemented in all regions and, if so, not always at the same time. However, decisions on key COVID-19 response measures were made at the federal level in both countries.

In Switzerland, the first COVID-19 response measure, a ban of events with more than 1000 visitors, was announced and implemented at the federal level on February 28 when there were some 25 confirmed COVID-19 cases (0.03 per 10,000 inhabitants) in Switzerland. Several measures at the cantonal level followed. For instance, many cantons introduced a ban on visits to retirement homes. Some 2.5 weeks after the first measure was implemented, the Federal Council decided to close all schools and childcare centers in Switzerland as well as non-essential shops, cafes, bars, and restaurants on March 16. In the following, we will refer to these measures as lockdown measures. At that point in time, the rate of confirmed infections in Switzerland was at 4.2 per 10,000 inhabitants. The schedule of response measures in the Principality of Liechtenstein (LI) was similar to that in Switzerland with the lockdown entering into force 2 days later. Due to the two countries’ similar schedules of COVID-19 response measures, their geographic proximity, and their economic, cultural, and political interconnection, we include LI as additional data point when investigating the impact of the lockdown measures in Switzerland.

In Germany, first measures at the federal level were implemented between March 9 and March 12. On March 8, when there were some 1000 reported COVID-19 cases (0.12 per 10,000 inhabitants) in Germany, the federal government advised against events with more than 1000 visitors. This recommendation was translated into a ban by most federal states, while others implemented it as recommendation only. As in Switzerland, schools and childcare centers in most German states closed on March 16; the remaining states followed within 2 days. The closure of all non-essential retailers, bars, and public events of any kind and the restriction of restaurant opening hours were decided at the federal level on March 16 when the overall rate of confirmed infections reached 1.1 per 10,000 inhabitants. The states implemented these measures between March 17 and March 20. Other than in Switzerland and LI, these measures were further enhanced later on. On March 22, a ban of groups with more than two individuals was decided at the federal level and several states additionally implemented curfews. Since April 17, more and more states have made wearing face masks in shops and public transport compulsory, resulting in a nationwide requirement to wear masks in public from April 27 on. Meanwhile, lockdown measures have been lifted gradually in Switzerland and Germany, with distinct schedules and exit strategies across countries and states. For instance, curfews ended in the respective German states around April 27, with the exception of Bavaria, where they ended on May 5. On May 6, a so-called emergency mechanism was put in place in Germany requiring counties to re-impose lockdown measures locally if the rate of new confirmed infections over 7 days exceeds 5 per 10,000 inhabitants.

## Data

For Switzerland and LI, data on confirmed COVID-19 infections as well as on COVID-19-related hospitalizations and deaths are amalgamated by the Swiss Federal Office of Public Health (FOPH) and made available to the interuniversity research consortium of the Swiss School of Public Health (www.ssphplus.ch). For each confirmed case, the FOPH gathers information on the reporting canton, test date, and patient’s age and gender from laboratory declarations. For our analysis, we aggregate the number of confirmed infections, hospitalizations, and fatalities by canton and test date; compute the respective cumulative numbers by canton and date; and complement the data with sociodemographic variables at the cantonal level (and for LI) from the statistical offices of Switzerland and LI. For each of the 26 Swiss cantons and LI, we calculate the rate of cumulative confirmed infections, hospitalizations, and fatalities per 10,000 inhabitants, as well as the median age of those tested positively for COVID-19 prior to the lockdown measures in Switzerland and LI. Furthermore, we construct indicators for whether a canton has introduced certain additional measures not imposed by the federal government along with variables providing the start date of such canton-level measures as stated in press releases of the respective cantons.

In Germany, all confirmed infections and deaths are reported to the Robert Koch Institute (RKI), a federal government agency and research institute for disease control and prevention. The RKI publishes data on the age group, gender, test date, and county of residence of each validated COVID-19 case reported to the institute. Only for the county of Berlin with 3.6 million inhabitants, the RKI also reports the urban residential district of confirmed cases. All in all, there are 401 counties in Germany and 12 residential districts in Berlin. Similar to Switzerland, we aggregate the data by county (or residential district, respectively) and test date, and compute cumulative confirmed cases and fatalities by county and date. We complement the data with sociodemographic variables at the county/district level from the Federal Office of Statistics, the statistical offices of the federal states, and the statistical office of the city of Berlin. As most measures in Germany were implemented at the state or even county level and at different points in time, we generate variables for all measures indicating whether and when they were imposed in each county.

**Fig. 1:**
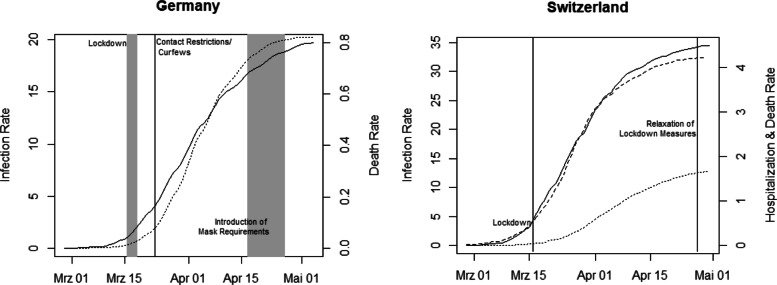
Cumulative confirmed infections (solid line), deaths (dotted line), and hospitalizations (dashed line) per 10,000 inhabitants in Germany and Switzerland

**Fig. 2:**
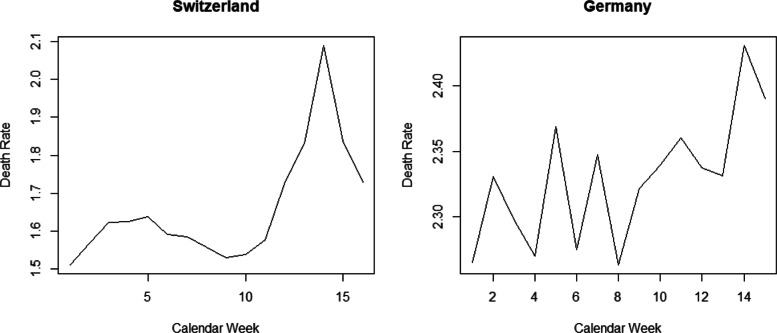
Overall deaths per 10,000 inhabitants by calendar week in Switzerland (left) and Germany (right). Source: federal statistical offices of Switzerland (www.bfs.admin.ch) and Germany (www.destatis.de); retrieval date: May 6

Figure 1 provides the cumulative numbers of confirmed COVID-19 infections and COVID-19-related deaths per 10,000 inhabitants in Germany (left) as well as cumulative numbers of confirmed infections, hospitalizations, and deaths in Switzerland (right). The figure suggests a flattening of the COVID-19 epidemic curve in both countries after the main COVID-19 measures have been in place for some weeks, which does, however, not necessarily exclusively reflect the causal impact of the measures. As a further descriptive statistic, Fig. 2 provides the overall deaths per 10,000 inhabitants (thus including COVID-19-related mortality) by calendar week in Germany and Switzerland since January 1, 2020 (provisional data). While the increase in mortality in March and April can be linked to the COVID-19 epidemic (a finding that also holds when controlling for the average mortality over 2015–2019), we cannot directly infer how large the increase would have been with and without the lockdown measures. For this reason, our analysis aims at shedding light on the causal effect of the measures.

## Econometric approach

In our analysis, we exploit the fact that the epidemic was more advanced in some regions than in others when the key control measures came into force. In Switzerland, for instance, Basel-Stadt had already more than 1 confirmed case per 10,000 inhabitants 12 days before the federal lockdown measures were implemented, while other cantons such as St. Gallen were at an earlier stage, reaching 1 confirmed infection per 10,000 inhabitants on the day of the lockdown. In Germany, the county of Heinsberg recorded more than 1 confirmed infection per 10,000 inhabitants already 19 days before the lockdown. In several other counties, this level of infections was reached only after the lockdown.

For Germany, we investigate the impact of the lockdown measures as well as the curfew on cumulative deaths per 10,000 inhabitants. For Switzerland and LI, we assess the causal effect of the lockdown on both cumulative hospitalizations and deaths per 10,000 inhabitants. The idea is to quantify the epidemic stage of each canton/county when measures were implemented by defining dates on which the health outcomes are measured relative to the day a canton/county first reached a certain rate of confirmed infections. For both Germany and Switzerland, we define the start date of the epidemic as the day when the rate of infections first reached or exceeded 1 infection per 10,000 inhabitants. In Switzerland, for instance, the start date of the epidemic in Basel-Stadt is on March 5 (late exposure to measures) while in St. Gallen the epidemic started on March 16 (early exposure to measures). Appendix 1 provides the start states for all Swiss cantons and LI.

Besides their obvious relevance for health care, a further motivation to consider hospitalization and death rates as outcomes is that their measurement is likely more robust to differences in testing strategies across regions than the measurement of confirmed COVID-19 infections. While the share of infections with mild symptoms being detected ceteris paribus likely rises with increased testing, the number of hospitalizations and fatalities gives a better estimate of the severeness of the epidemic in terms of human loss and strains for the health care system. As both Germany and Switzerland maintain a system of mandatory health insurance and neither country generally saw their hospitalization capacities exhausted, we would suspect that the number of COVID-19-related hospitalizations in general mirrors well the number of individuals infected with COVID-19 that are in need of hospitalization. Nevertheless, a potential concern in our analysis is that the criteria for hospitalizations might not be uniform across regions. The same may apply to the measurement of fatalities, i.e., the definition of criteria according to which a decease is attributed to COVID-19. If such measurement issues in health outcomes are not systematically associated with the region-specific start date of the epidemic (or more generally, with the policy interventions considered), they do not bias the results of our analysis. However, if for instance regions with an earlier start date and a more advanced epidemic systematically applied more stringent rules for hospital admissions (e.g., to prevent capacity constraints), this could also entail an underestimation of COVID-19 fatalities due to underreporting deceases at home. In this case, our analysis of the relative timing of measures presented below would likely provide a lower bound of the true effect on (capacity-unconstraint) hospitalizations and fatalities.

### OLS approach

We compare the average number of cumulative hospitalizations and fatalities per 10,000 inhabitants on canton/county-specific epidemic days across three groups of cantons/counties. These groups are defined by the canton/county-specific epidemic day when lockdown measures came into place. For Switzerland and LI, we distinguish the groups of cantons as follows. Cantons reaching or exceeding 1 confirmed infection per 10,000 inhabitants at most 4 days before the lockdown measures are exposed to the measures at a relatively early stage of the epidemic and constitute the reference group (sample size *N*=8). Those cantons with at least 1 confirmed infection per 10,000 inhabitants between 5 and 8 days before March 16 (or March 18 in the case of LI) are the intermediate intervention group (*N*=11). Those with a canton-specific start date at least 9 days before March 16 are the late intervention group (*N*=8).

For Germany, we proceed analogously and define the treatment groups based on the days between the county-specific start of the epidemic and the lockdown according to the retail closures between March 17 and 20, but with somewhat different time brackets. Counties with at least 1 confirmed infection per 10,000 inhabitants not earlier than 3 days after the implementation of lockdown measures make up the reference group. The specified start dates are later than the lockdown, which may at first glance raise endogeneity concerns. However, any effect of the measures can materialize in the outcomes only with a substantial time lag of more than 1.5 weeks (due to incubation time and reporting lags), as also confirmed in our analysis. Therefore, confirmed infection rates are not yet influenced by the measures even several days after the lockdown. Yet, we exclude 4 counties having start dates as late as 9 days after the lockdown or later, leaving us with a reference group of *N*=52. The intermediate intervention group is comprised of all counties with at least 1 confirmed infection per 10,000 inhabitants between 3 days before and 2 days after the lockdown (*N*=275). The late intervention group consists of counties with at least 1 confirmed infection per 10,000 inhabitants more than 3 days before the lockdown (*N*=81).

We estimate the difference in cumulative death rates, as well as hospitalization rates for Switzerland and LI, between either of the two treatment groups (intermediate and late intervention group) and the reference group by means of an OLS regression with treatment indicators. We also control for the following canton/county-specific covariates: population size and density, income per capita, age distribution, age structure of positively tested up to the lockdown, the initial canton/county-specific growth trend for confirmed cases, and canton-specific bans on visits in hospitals and retired homes entering into force prior to the lockdown. The large number of counties in Germany allows us to further control for past mortality by age group, past mortality rate related to respiratory diseases, and hospital capacities (beds/1000 inhabitants). We also control for state-specific measures entering into force prior to the general lockdown, like bans of or recommendations against events with more than 1000 visitors, as well as curfews imposed in some states only a few days after the general lockdown. Appendix 2 provides descriptive statistics of the covariates used in the analysis of the German and Swiss measures for the respective total samples as well as separately for the various intervention groups.

Though aiming to control for confounders jointly affecting the region-specific epidemic and the health outcomes in a comprehensive way, we cannot completely rule out that some important characteristics are omitted in our analysis. For instance, we cannot directly control for the amount of intergenerational interactions, which is according to Bayer and Kuhn (2020) correlated with the ratio of deaths over confirmed cases and could potentially differ across regions. We, however, point out that the results for the relative timing of measures are quite robust to (not) controlling for covariates. Since the lockdown measures in Germany and in Switzerland have been eased starting with April 20 and April 27, respectively, we evaluate the effect of the relative timing of measures on the health outcomes in these countries until April 23 and April 30, respectively.

For Germany, we also investigate the impact of curfews, as introduced in some federal states between March 21 and 23 on top of the federally imposed contact restriction that banned groups of more than 2 individuals. The OLS regression contains a binary treatment indicator for curfews as well as a range of control variables. The latter include the previously mentioned county-specific characteristics, growth trends and COVID-19 response measures, and in addition the cumulative confirmed infections and death rates on several days prior to the curfews, in order to make regions exposed and not exposed to curfews as similar as possible. The OLS specification is provided in Appendix 3, and descriptive statistics for counties with and without curfews in Appendix 2.

### Doubly robust estimation

The larger number of regions in Germany allows us to also consider a more flexible (so-called semiparametric) evaluation approach based on doubly robust (DR) estimation, see Robins et al. (1994) and Robins and Rotnitzky (1995). It is based on (i) estimating a logit model for the treatment probability as a function of the covariates as well as a linear model for the outcome as a function of the treatment and the covariates and (ii) using the respective model predictions as plug-in parameters for the estimation of the treatment effects. DR provides consistent effect estimates if at least one of the plug-in models is correctly specified and thus relies on less stringent assumptions than OLS. Using the “drgee” package of Zetterqvist and Sjölander (2015) for the statistical software “R,” we apply DR for estimating the average effect of a binary intervention separately to subsets of counties consisting of the reference group and either the intermediate intervention group or the late intervention group.

### Synthetic control approach

For Switzerland, we complement the regression analysis with a synthetic control approach, a quantitative case study method suggested in Abadie and Gardeazabal (2003). To this end, we compare cumulative hospitalization and fatality rates in a specific canton with a late exposure to the lockdown to the rates of an artificially (or synthetically) created counterfactual canton. This synthetic canton should be comparable to the original reference canton in terms of covariates outlined in Section 4.1 and pre-treatment health outcomes (measured 2 and 5 days after the start date), but characterized by an earlier exposure to the lockdown.^2^ To this end, the synthetic canton is generated as a weighted average of control cantons with an earlier exposure using the “Synth” package of Abadie et al. (2011) for the statistical software “R,” where the weights depend on how close their characteristics and pre-treatment outcomes match the values of the reference canton with the later exposure. The control pool includes all in all 11 cantons that reached 1 confirmed infection per 10,000 inhabitants at most 3 days before the lockdown.

## Results

### Germany

**Fig. 3:**
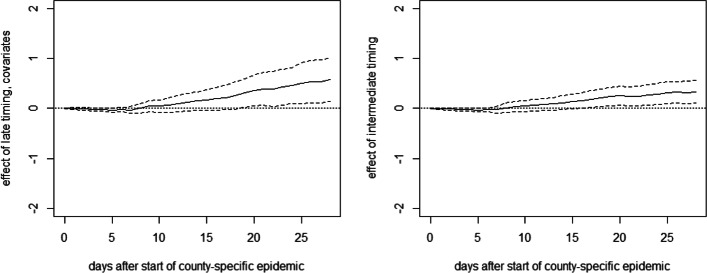
OLS effects of late (left) and intermediate (right) timing of measures on cumulative deaths per 10,000 inhabitants in Germany

Figure 3 reports the mean differences in cumulative fatalities per 10,000 inhabitants between either treatment group and the early intervention group (reference group) per day up to 28 days after the county-specific start date (solid lines) based on the OLS approach.^3^ It also includes 90% confidence intervals (dashed lines). The mean differences in fatality rates between the late and the early intervention groups (left) remain close to zero during the first 2.5 weeks of the county-specific epidemic but show a positive and statistically significant tendency thereafter. The point estimates suggest that after 1 month, fatalities per 10,000 inhabitants are reduced by 0.6 cases under an earlier lockdown. Also, the difference in death rates between the intermediate and the early intervention groups is statistically significant at the 10% level, but (expectedly) smaller in magnitude. Overall, the results suggest that the relative timing of measures had a perceptible impact on COVID19-related fatalities in Germany. We note that Appendix 3 provides the OLS specification with the full list of coefficients on treatments and covariates along with standard errors 28 days after the start of the epidemic. Concerning the robustness of our findings, we note that estimations without controlling for observed covariates yield qualitatively similar results, see Appendix 4.

**Fig. 4:**
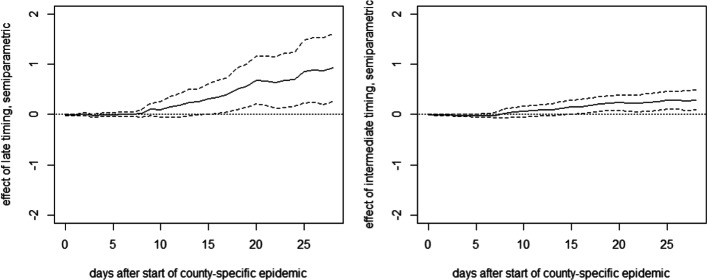
DR effects of late (left) and intermediate (right) timing of measures on cumulative deaths per 10,000 inhabitants in Germany

Figure 4 reports the estimates of DR, which are generally similar to OLS, though suggesting an even stronger effect of a late timing of lockdown measures on the death rate. The point estimate suggests that an earlier lockdown reduces fatalities by roughly 1 case per 10,000 1 month after the start of the epidemic.

With 27% of the German population living in counties with late lockdown timing, a rough back-of-the-envelope calculation based on the OLS point estimates suggests that some 1283 COVID-19-related deaths (2080 when using the DR results) could have been prevented in Germany over the first 4 weeks after lockdown implementation if the counties with late timing had implemented the lockdown early, meaning no later than 3 days before reaching or exceeding the level of 1 confirmed infection per 10,000 inhabitants. If all 275 states with intermediate lockdown timing had implemented the lockdown early, the death toll could have been further reduced by some 1816 (1580 based on DR results).

**Fig. 5:**
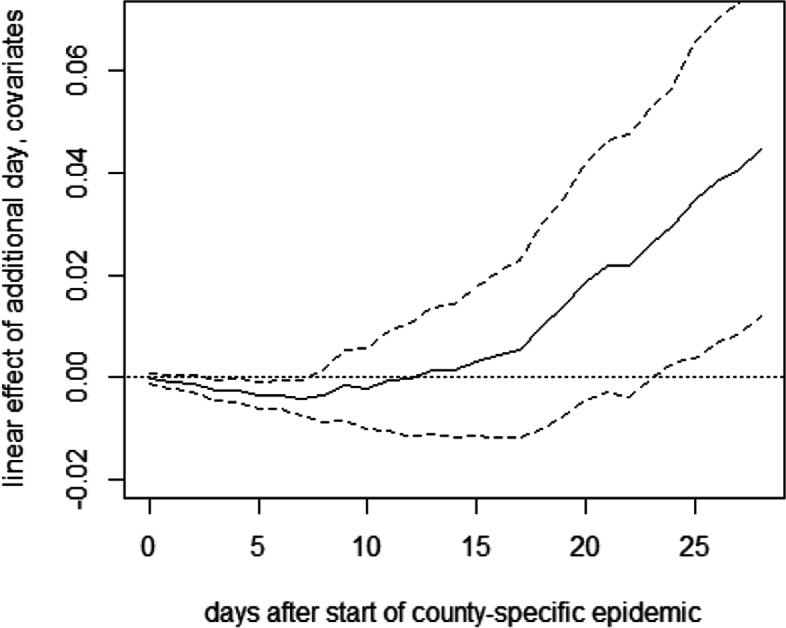
OLS effect of delaying lockdown by 1 day on deaths per 10,000 inhabitants in Germany

Figure 5 reports the results of a further OLS regression, in which the treatment indicators for the intermediate and late intervention groups are replaced by the time lag between the county-specific start date of the epidemic and the lockdown, in order to (linearly) estimate the effect of the lag. This can be interpreted as the average effect of waiting an additional day before implementing the measures. The point estimates suggest that each additional day without lockdown entails on average 0.04 to 0.05 additional fatalities per 10,000 inhabitants after 1 month of the epidemic, even though the confidence intervals are rather wide (but yet do not include a zero effect). Again, these results are quite robust to not controlling for covariates, see Appendix 4.

Our results also appear interesting with respect to one key element in the German exit strategy, the so-called emergency mechanism requiring counties to re-impose lockdown measures locally if the rate of new confirmed infections over 7 days exceeds 5 per 10,000 inhabitants. Though the local epidemic start date is based on the cumulative rate of confirmed infections and the threshold of the German policy is based on the 7-day running infection rate, one may want to assess the appropriateness of this threshold in the light of our findings about the importance of lockdown timing. In fact, the threshold for re-implementing lockdown measures can be regarded as late rather than intermediate or early intervention with respect to our definition, which seems worth considering given the threat of a second wave. However, the situation during the early phase of the epidemic is most likely not comparable to that in a later point in time, where the hope is that larger testing capacities and better policy response lead to an earlier detection and containment of local COVID-19 outbreaks and that the increased awareness in the population entails an adoption of social distancing and hygiene measures that sufficiently slow down the transmission.

**Fig. 6:**
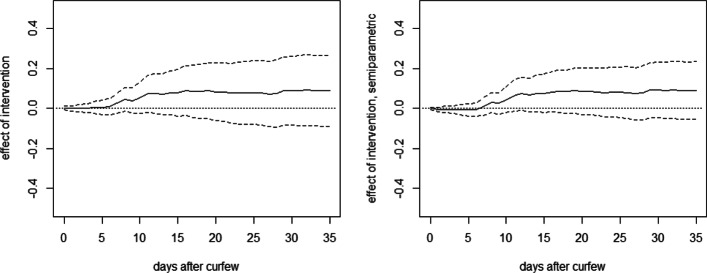
OLS (left) and DR (right) effects of curfews on deaths per 10,000 inhabitants in Germany

Furthermore, the left graph in Fig. 6 provides the OLS-based effects of curfews relative to contact restrictions, i.e., bans of gatherings with more than 2 persons, under all other lockdown measures already in place. The estimates have a positive sign, which appears counterintuitive as curfews are more restrictive than contact restrictions, but are never statistically significantly different from zero throughout the evaluation window which starts on March 23 and ends 35 days later. The same finding applies to estimation results based on DR, which are shown in the right graph of Fig. 6. Therefore, we do not find evidence that curfews are more effective than banning groups for reducing fatality rates.

### Switzerland and LI

**Fig. 7:**
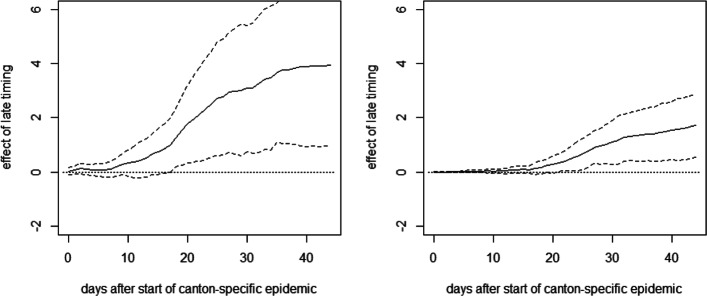
Effect of late timing of measures on cumulative hospitalizations (left) and deaths (right) per 10,000 inhabitants

Figure 7 reports the OLS estimates of the mean differences in cumulative hospitalizations (left) and fatalities (right) per 10,000 inhabitants between the late and the early intervention groups up to 44 days after the start of the canton-specific epidemic (solid line), as well as 90% confidence intervals (dashed lines). See Appendix 3 for the full OLS specification with the coefficients on treatments and covariates on the last day of the evaluation window and fatalities as outcome variable.

We note that the canton of Ticino is excluded from this analysis due to its comparably strong economic and social ties with Northern Italy (which was particularly severely affected by the COVID19 crisis), as this could arguably have affected the canton’s hospitalizations and fatalities. However, our findings are quite similar when including Ticino in the regression, as well as when not controlling for covariates, see Appendix 5.

**Fig. 8:**
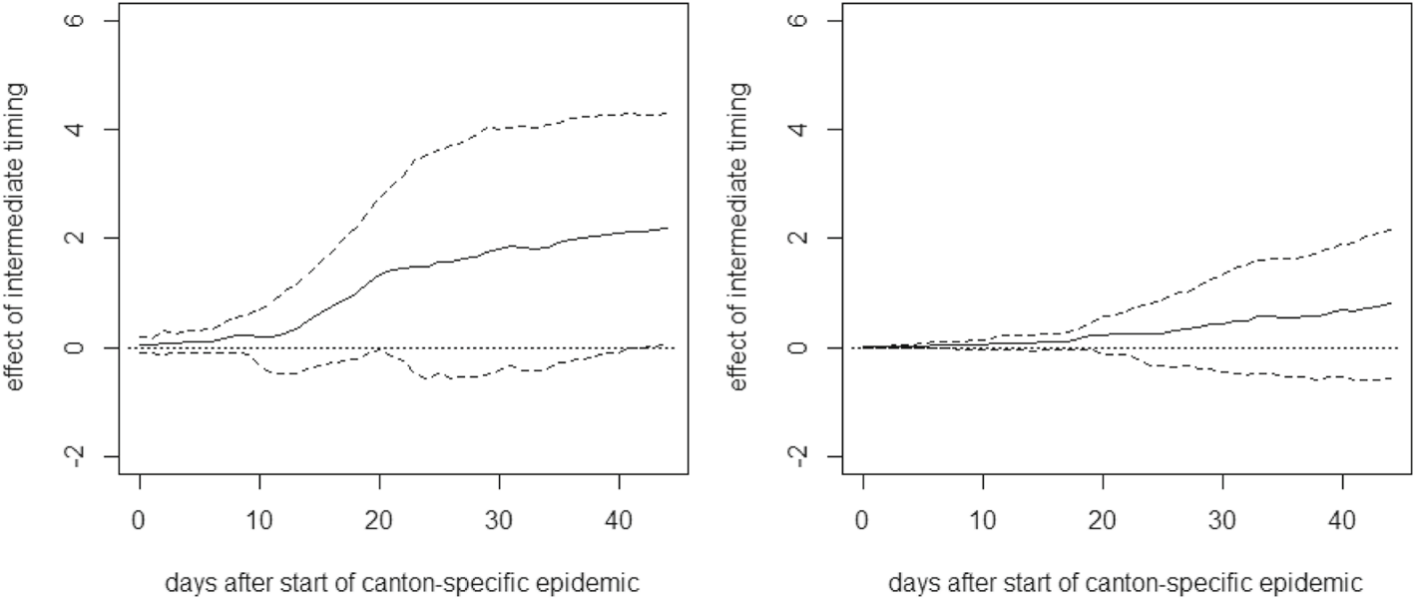
Effect of intermediate timing of measures on cumulative hospitalizations (left) and deaths (right) per 10,000 inhabitants

As for Germany, we see no immediate effect of the relative timing of measures on the health outcomes right after their introduction. However, after about 2 weeks, there is a positive tendency in the effect on cumulative hospitalizations that becomes statistically significant at the 10% level about 2.5 weeks after the start of the canton-specific epidemic. The point estimates suggest that after 1.5 months, cumulative hospitalizations per 10,000 inhabitants increase by almost 4 cases when introducing the measures later rather than earlier, even though the estimates are not very precise (i.e., confidence intervals are wide). A qualitatively similar pattern is observed for the effect on cumulative deaths, which becomes statistically significant after about 3 weeks. The point estimates suggest an increase of 1 to 2 fatalities per 10,000 inhabitants in the case of a later lockdown, but precision is again low. Figure 8 reports the same analysis for a comparison of the groups with intermediate and early timing. As these two groups are more similar in terms of the relative timing of the measures, differences are less pronounced and never statistically significant in all but one case, which might be due to low statistical power related to the small number of cantons.^4^

A rough back-of-the-envelope estimation based on these point estimates suggests that some 333 COVID-19-related deaths and some 764 hospitalizations could have been prevented during the time of the lockdown in Switzerland if the cantons with late timing had implemented the lockdown at most 4 days after reaching or exceeding the level of 1 confirmed infection per 10,000 inhabitants.

**Fig. 9:**
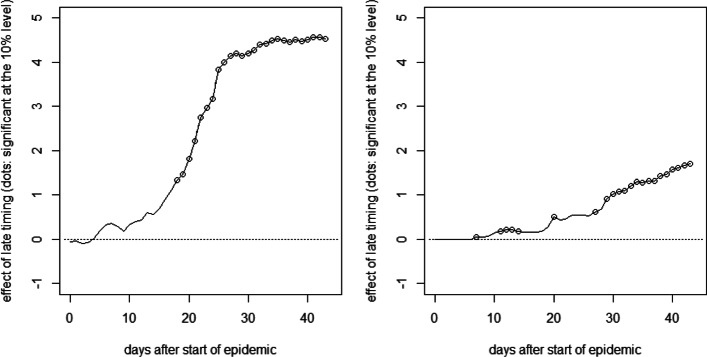
Effect of late timing of measures on cumulative hospitalizations (left) and deaths (right) per 10,000 inhabitants in Basel-Stadt

Finally, we report the results of the synthetic control method for two cantons experiencing the lockdown rather late relative to their start date of the epidemic. Figure 9 plots the difference in cumulative hospitalizations (left) and deaths (right) per 10,000 inhabitants on a daily base after the canton-specific start date between Basel-Stadt, which was on day 12 of the epidemic when the measures came into force, and its synthetic counterfactual. The latter is generated from a control group of 11 cantons with an earlier timing (with start dates between 3 days before and 1 day after the lockdown). Dots on the solid line imply that the differences are statistically significant at the 10% level according to placebo tests in the control group, in which each of the 11 cantons is considered as (pseudo-)treated in a rotating scheme in order to estimate its (pseudo-)counterfactual based on the remaining 10 cantons. We, however, note that the estimation of *p* values might be imprecise, due to the low number of control cantons available for the placebo tests.

Again, the relative timing of measures shows no immediate effect on hospitalizations but the difference becomes statistically significant after roughly 2.5 weeks. The point estimates suggest that the hospitalization rate in Basel-Stadt could have been reduced by more than 4 hospitalizations if the lockdown measures had been introduced earlier. Similarily, the fatalities per 10,000 inhabitants could have been reduced by 1 to 2 cases about 1.5 months after the start of the epidemic. As for the OLS analysis, the exact numbers should, however, be interpreted with caution, as they are imprecisely estimated and canton-specific factors not considered in the analysis could play a role as well.

**Fig. 10:**
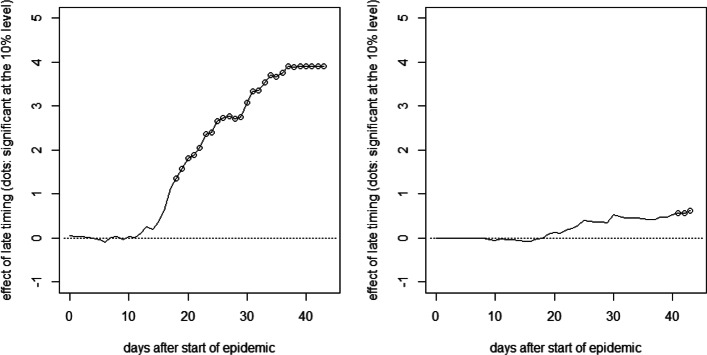
Effect of late timing of measures on cumulative hospitalizations (left) and deaths (right) per 10,000 inhabitants in Neuchâtel

Figure 10 reports the results for Neuchâtel, another canton with a relatively late timing, which was on day 10 of the epidemic when the measures came into force. Concerning the effect of the lockdown timing on hospitalizations, we find a similar pattern as for Basel-Stadt. Albeit the effect on COVID-19-related fatalities is somewhat less pronounced, it turns statistically significant in the final periods of the evaluation window.

## Conclusion

In this paper, we analyzed the impact of lockdown timing on COVID-19-related fatalities and hospitalizations in Germany and Switzerland. For doing so, we exploited the fact that measures differed across regions and that the epidemic was more advanced in some regions than in others when certain measures came into force. Using OLS and doubly robust estimation, we compared the development of COVID-19-related hospitalization and death rates—two indicators which are arguably rather robust to regional differences in COVID-19 testing policies—across regions that have been at different epidemic stages when exposed to the lockdown measures. For Switzerland, we also applied a synthetic control approach to investigate the impact of the relative timing of the lockdown in two selected cantons. In addition, we analyzed the impact of curfews as implemented in some German states on top of the federal ban on gatherings of more than 2 persons based on a cross-regional comparison.

For both countries, we found an earlier lockdown to be more effective than a later one, as cumulative hospitalization and fatality rates measured relative to the region-specific start date of the epidemic were higher in regions with a more advanced spread of COVID-19 when the measures came into force. In contrast, our results did not provide evidence for curfews being more effective than bans on gatherings under the other lockdown measures already in place.

## Appendix 1. Start dates of canton-specific epidemics

**Table 1:** 2020 dates on which 1 confirmed infection per 10,000 inhabitants was reached in the Swiss cantons and LI

Canton	Start date
Aargau (AG)	March 16
Appenzell Innerrhoden (AI)	March 13
Appenzell Ausserrhoden (AR)	March 13
Bern (BE)	March 14
Basel-Landschaft (BL)	March 11
Basel-Stadt (BS)	March 05
Fribourg (FR)	March 11
Genève (GE)	March 09
Glarus (GL)	March 12
Graubünden (GR)	March 09
Jura (JU)	March 10
Luzern (LU)	March 16
Neuchâtel (NE)	March 07
Nidwalden (NW)	March 09
Obwalden (OW)	March 11
St. Gallen (SG)	March 16
Schaffhausen (SH)	March 17
Solothurn (SO)	March 16
Schwyz (SZ)	March 12
Thurgau (TG)	March 16
Ticino (TI)	March 05
Uri (UR)	March 17
Vaud (VD)	March 09
Valais (VS)	March 12
Zug (ZG)	March 13
Zürich (ZH)	March 12
Principality of Liechtenstein (LI)	March 09

## Appendix 2. Descriptive statistics of covariates

**Table 2:** Mean of covariates considered in the estimations using the German data in the total sample, the late intervention group, the intermediate intervention group, and the early intervention group, respectively

Variable	Total sample	Late timing	Intermediate timing	Early timing	Curfew	No curfew
	*N*=408	*N*=81	*N*=275	*N*=52	*N*=149	*N*=259
Population	203,103	276,529	197,295	119,444	158,786	228,598
Population density	671	929	665	301	440	804
Income per capita (euro)	37,224	41,686	36,505	34,076	38,325	36,591
Share of population aged 65+	0.222	0.208	0.221	0.244	0.226	0.219
80+ mortality rate (per 1000 inhabitants), 2017	6.52	5.96	6.52	7.36	6.68	6.42
Share of respiratory disease-related deaths, 2016	0.07	0.069	0.071	0.067	0.066	0.072
Hospital beds per 1000 inhabitants	6.31	6.08	6.25	6.97	6.69	6.09
Share of confirmed infections aged 80+ prior to lockdown	0.019	0.024	0.018	0.014	0.022	0.017
Initial growth trend for confirmed cases in log points	0.209	0.23	0.234	0.049	0.185	0.224
Ban of events with > 1000 participants	0.917	0.889	0.924	0.923	1	0.869
Curfew	0.365	0.247	0.378	0.481	1	0
Ban of groups of > 5 persons (prior to contact ban/curfew)	0.223	0.21	0.236	0.173	0	0.351
Permission to meet with 1 non-household member	0.711	0.802	0.698	0.635	0.262	0.969

**Table 3:** Means of covariates considered in the estimations using the Swiss (and LI) data in the total sample, the late intervention group, the intermediate intervention group, the early intervention group, the group of counties with curfew, and the group of counties without curfew, respectively

Variable	Total sample	Late timing	Intermediate timing	Early timing
	*N*=27	*N*=8	*N*=11	*N*=8
Population	315,648	286,649	268,466	409,524
Population density	503	1046	278	271
Income per capita (CHF)	80,404	102,840	73,134	67,964
Share of population aged 65+	0.192	0.193	0.19	0.193
Median age of confirmed infections prior to lockdown	50.19	49.56	49.09	52.31
Initial growth trend of confirmed cases in log points	0.235	0.239	0.21	0.266
Ban on visits to retirement homes	0.593	0.5	0.727	0.5

## Appendix 3. OLS specifications for Germany and Switzerland

**Table 4:** OLS estimates for Germany 28 days after the start of the county-specific epidemic with fatalities per 10,000 inhabitants as outcome variable

	Estimate	Standard error
Intercept	−1.1628	0.6661
Intermediate timing	0.3348	0.1403
Late timing	0.5729	0.2663
Share of population aged 65+	−6.4132	2.8281
Population: 0–105,878	0.4388	0.2112
Population: 105,879–158,080	0.2848	0.135
Population: 158,081–251,534	0.0665	0.0985
Population density: 0–117.3	0.0801	0.1425
Population density: 117.3–206.7	0.1201	0.1454
Population density: 206.7–779.7	0.0613	0.1347
Income per capita: 0–27,934	−0.1437	0.1561
Income per capita: 27,935–33,109	−0.1721	0.1439
Income per capita: 33,110–40,506	0.0568	0.1749
Share of confirmed infections aged 80+ prior to lockdown	4.4466	2.1463
80+ mortality rate (per 1000 inhabitants), 2017	0.2066	0.091
Share of respiratory disease-related deaths, 2016	0.9538	3.7197
Hospital beds per 1000 inhabitants	−0.0329	0.0184
Initial growth trend for confirmed cases in log points: 0–0.14	−0.1188	0.1852
Initial growth trend for confirmed cases in log points: 0.14–0.21	−0.089	0.1407
Initial growth trend for confirmed cases in log points: 0.21–0.28	−0.0369	0.136
Confirmed infections per 10,000 inhabitants on epidemic day 4	0.2556	0.0805
Recommendation against events with > 1000 visitors	0.1594	0.0985
Ban of events with > 1000 visitors	0.7132	0.141
Curfew	0.2403	0.1111

**Table 5:** OLS estimates for Switzerland and LI 44 days after the start of the canton-specific epidemic with fatalities per 10,000 inhabitants as outcome variable

	Estimate	Standard error
Intercept	39.2105	45.0916
Intermediate timing	0.7961	0.7712
Late timing	1.7187	0.6681
Share of population aged 65+	−337.2691	362.2737
Squared share of population aged 65+	848.3766	950.0775
Population: 0–59,999	−0.5647	1.1326
Population density	4e −04	4e −04
Income per capita	0	0
Median age of confirmed infections prior to lockdown	−0.2783	1.308
Squared median age of confirmed infections	0.003	0.0131
Initial growth trend for confirmed cases in log points	6.3784	8.0649
Confirmed infections per 10,000 inhabitants on epidemic day 4	0.0172	0.6938
Ban on visits to retirement homes	0.153	0.4966

**Table 6:** OLS estimates for the impact of curfews (compared to contact restrictions) 35 days after the imposition of curfews with fatalities per 10,000 inhabitants as outcome variable

	Estimate	Standard error
Intercept	−0.5481	0.5532
Curfew	0.089	0.1081
Share of population aged 65+	−5.6962	2.9762
Income per capita: 0–27,934	−0.0998	0.1872
Income per capita: 27,935–33,109	−0.0444	0.1598
Income per capita: 33,110–40,506	−0.056	0.1298
Population density: 0–117.3	0.0077	0.1547
Population density: 117.3–206.7	0.1532	0.1558
Population density: 206.7–779.7	0.0388	0.1315
Population: 0–105,878	0.1964	0.1917
Population: 105,879–158,080	0.1198	0.1565
Population: 158,080–251,534	−0.048	0.1067
Share of confirmed infections aged 80+	0.6616	2.0497
80+ mortality rate (per 1000 inhabitants), 2017	0.2029	0.0692
Share of respiratory disease-related deaths, 2016	3.5314	3.6582
Hospital beds per 1000 inhabitants	−0.0201	0.016
Confirmed fatalities per 10,000 inhabitants 10 days before curfew	−4.3731	4.1915
Confirmed fatalities per 10,000 inhabitants 5 days before curfew	−2.7013	3.4591
Confirmed fatalities per 10,000 inhabitants 4 days before curfew	1.3937	3.7116
Confirmed fatalities per 10,000 inhabitants 3 days before curfew	−2.8829	3.6353
Confirmed fatalities per 10,000 inhabitants 2 days before curfew	5.058	2.5642
Confirmed fatalities per 10,000 Inhabitants 1 day before curfew	2.1268	2.1477
Confirmed cases per 10,000 inhabitants 25 days before curfew	2.478	4.2755
Confirmed cases per 10,000 inhabitants 20 days before curfew	0.9095	1.5009
Confirmed cases per 10,000 inhabitants 15 days before curfew	0.0804	0.4324
Confirmed cases per 10,000 inhabitants 10 days before curfew	−0.3862	0.2614
Confirmed cases per 10,000 inhabitants 5 days before curfew	0.0339	0.2059
Confirmed cases per 10,000 inhabitants 4 days before curfew	−0.3237	0.3682
Confirmed cases per 10,000 inhabitants 3 days before curfew	0.1382	0.3992
Confirmed cases per 10,000 inhabitants 2 days before curfew	−0.148	0.2767
Confirmed cases per 10,000 inhabitants 1 day before curfew	0.3193	0.2064
Initial growth trend for confirmed cases in log points	0.0158	0.0264
Recommendation against events with > 1000 visitors	0.2291	0.075
Ban of events with > 1000 visitors	0.6488	0.1937
Ban of groups of > 5 persons (prior to contact ban/curfew)	0.1391	0.1265
Permission to meet with 1 non-household member	−0.1802	0.1271

## Appendix 4. Estimations for Germany without covariates

**Fig. 11:**
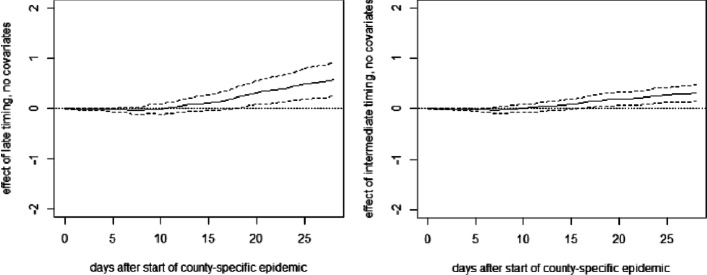
OLS effects of late (left) and intermediate (right) timing of measures on cumulative deaths per 10,000 inhabitants without covariates

**Fig. 12:**
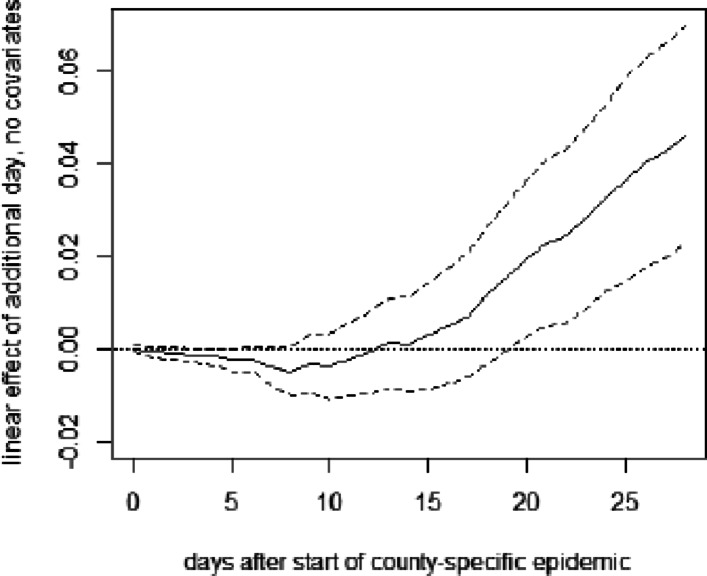
OLS effect of delaying lockdown by 1 day on deaths per 10,000 inhabitants in Germany without covariates

## Appendix 5. Estimations for Switzerland without covariates and including Ticino

**Fig. 13:**
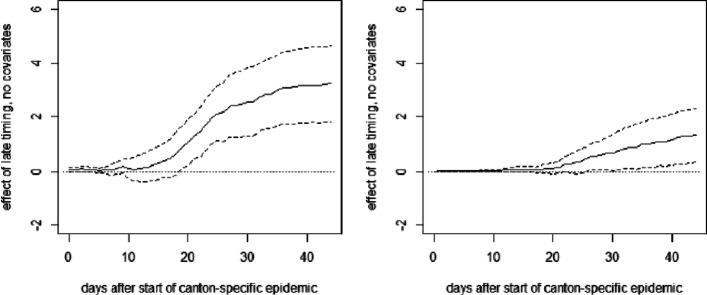
OLS effect of late timing of measures on cumulative hospitalizations (left) and deaths (right) per 10,000 inhabitants without covariates excluding Ticino

**Fig. 14:**
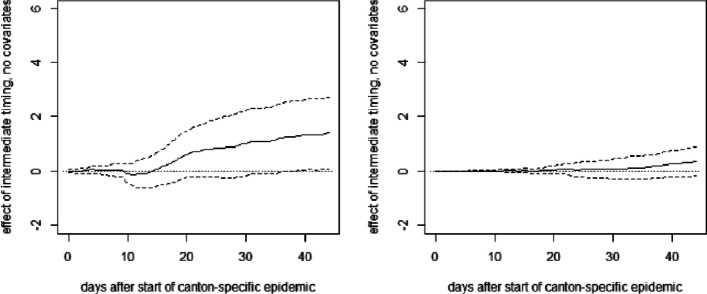
OLS effect of intermediate timing of measures on cumulative hospitalizations (left) and deaths (right) per 10,000 inhabitants without covariates excluding Ticino

**Fig. 15:**
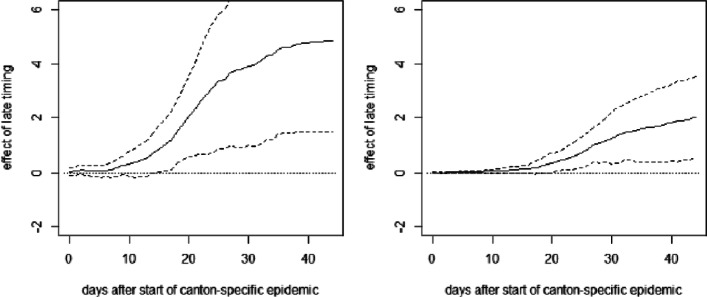
OLS effect of late timing of measures on cumulative hospitalizations (left) and deaths (right) per 10,000 inhabitants with covariates including Ticino

**Fig. 16:**
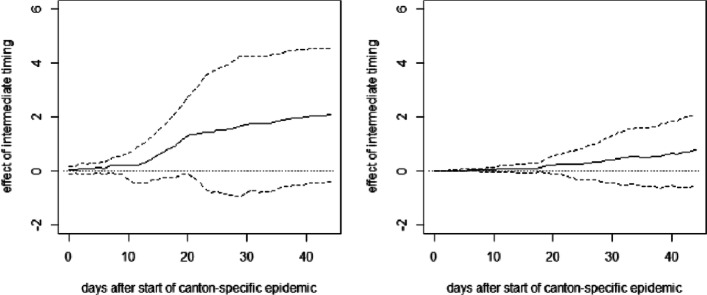
OLS effect of intermediate timing of measures on cumulative hospitalizations (left) and deaths (right) per 10,000 inhabitants with covariates including Ticino

## Data Availability

The data on confirmed infections, hospitalizations, and deaths in Switzerland is available to member universities of the interuniversity research consortium of the Swiss School of Public Health (www.ssphplus.ch). Data on confirmed infections and deaths in Germany is published by the Robert Koch Institute (https://npgeo-corona-npgeo-de.hub.arcgis.com). The sociodemographic data is published by the Swiss and German federal offices of statistics, the statistical office of Liechtenstein, the statistical offices of the German federal states, and the statistical office of the city of Berlin.

## Footnotes

1 The estimated effect on fatalities is also negative but less precise.

2 In contrast to the OLS specification provided in Appendix 3, squared variables (i.e., the squares of the population share aged 65+ and of the median age of confirmed infections prior to the lockdown) are not included. In addition, the dummy for the number of inhabitants being smaller than 60,000 is replaced by the actual number of inhabitants.

3 The motivation for the 28-day window is that we would like to include all (but 4) counties while at the same time only considering the period when the lockdown measures were fully implemented. As the last county we include in our evaluation sample saw its start of the epidemic 8 days after the lockdown, the time range considered in the analysis is limited to this specific window not including any effects of the first easing of lockdown measures starting with April 20.

4 For cumulative fatalities, we also run the OLS regression using an alternative data source based on calculations of the statistics office of the canton of Zurich, available at https://statistik.zh.ch(retrieved on May 15). We obtain a comparable pattern. Namely, the late intervention effect turns statistically significant after about 3 weeks with even somewhat higher point estimates (approaching 3) at the end of the evaluation window. The intermediate intervention effect is again insignificant.
